# Multiple origins of melanism in two species of North American tree squirrel (*Sciurus*)

**DOI:** 10.1186/s12862-019-1471-7

**Published:** 2019-07-11

**Authors:** Helen R. McRobie, Nancy D. Moncrief, Nicholas I. Mundy

**Affiliations:** 1 School of Life Sciences, Anglia Ruskin University, Cambridge, CB1 1PT UK; 2 Virginia Museum of Natural History, Martinsville, VA 24112 USA; 3 Department of Zoology, University of Cambridge, Cambridge, CB2 3EJ UK

**Keywords:** Adaptive introgression, Melanism, Melanocortin-1-receptor, Agouti signalling protein, *Sciurus carolinensis*, *Sciurus niger*, Convergent evolution

## Abstract

**Background:**

While our understanding of the genetic basis of convergent evolution has improved there are still many uncertainties. Here we investigate the repeated evolution of dark colouration (melanism) in eastern fox squirrels (*Sciurus niger;* hereafter “fox squirrels”) and eastern gray squirrels (*S. carolinensis;* hereafter “gray squirrels”).

**Results:**

We show that convergent evolution of melanism has arisen by independent genetic mechanisms in two populations of the fox squirrel. In a western population, melanism is associated with a 24 bp deletion in the melanocortin-1-receptor gene (*MC1RΔ24* allele), whereas in a south-eastern population, melanism is associated with a point substitution in the agouti signalling protein gene causing a Gly121Cys mutation*.* The *MC1R∆24* allele is also associated with melanism in gray squirrels, and, remarkably, all the *MC1R∆24* haplotypes are identical in the two species. Evolutionary analyses show that the *MC1R∆24* haplotype is more closely related to other *MC1R* haplotypes in the fox squirrel than in the gray squirrel. Modelling supports the possibility of gene flow between the two species.

**Conclusions:**

The presence of the *MC1R∆24* allele and melanism in gray squirrels is likely due to introgression from fox squirrels, although we cannot completely rule out alternative hypotheses including introgression from gray squirrels to fox squirrels, or an ancestral polymorphism. Convergent melanism in these two species of tree squirrels has evolved by at least two and probably three different evolutionary routes.

**Electronic supplementary material:**

The online version of this article (10.1186/s12862-019-1471-7) contains supplementary material, which is available to authorized users.

## Background

The origin of adaptive genetic variation is one of the key issues in evolutionary biology. Such variation generally depends on new mutations or standing variation. Another less well understood means of adaptation is adaptive introgression where interspecific mating occurs followed by generations of backcrossing and selection for advantageous introgressed alleles. Hybridisation between closely related species has been widely documented, but the role of such hybridisation in adaptation is not always clear. Adaptive introgression has been recognised for some time as an important source of genetic variation in plants, for example between sunflower species [1], between iris species [2], and between ragwort and groundsel [3]. Until recently, there were fewer convincing examples in animals, an early case being between species of Australian fruit fly [4]. More recent examples include an allele at the *K* locus leading to melanism that introgressed from domestic dogs to wolves [5], the *vkorc1* allele that confers resistance to rat poison among Old World mice [6], variation at *agouti* (*ASIP*) associated with winter coat colour in snowshoe hares [7], alleles that affect beak shape in Darwin’s finches [8, 9], and loci controlling colour patterns in Heliconius butterflies [10].

Colouration in animals has a wide range of adaptive functions including concealment, signalling, protection and thermoregulation [11]. Melanism (darkened colouration) is found in many diverse species and two of its major functions are to provide camouflage from predators, e.g. in lizards [12] and rock pocket mice [13] and to give a thermal advantage, e.g. in butterflies, ladybirds, snails and snakes [10, 14]. In amniote vertebrates, variation in dark colouration is primarily caused by variation in the amount of black/brown eumelanin present. Of the more than 300 loci which control melanin pigmentation in vertebrates [15], two key interacting loci have been found to be repeatedly involved in adaptive variation in melanin colouration: the melanocortin-1 receptor (*MC1R*) gene and agouti signalling protein (*ASIP*) gene. High activity of the MC1R protein leads to enhanced synthesis of eumelanin, a process that is inhibited by ASIP, so that gain-of-function mutations of *MC1R* or loss-of-function mutations in *ASIP* lead to melanism [16, 17]. In wild populations, mutations in *MC1R* have been associated with melanism in lizards [12], ~ 10 species of birds [18, 19] and a variety of mammals, from rodents [13] to cats [20, 21]. Mutations in *ASIP* have been associated with melanism in birds [22], rodents [23], hares [7] and cats [21, 24].

**Fig. 1:**
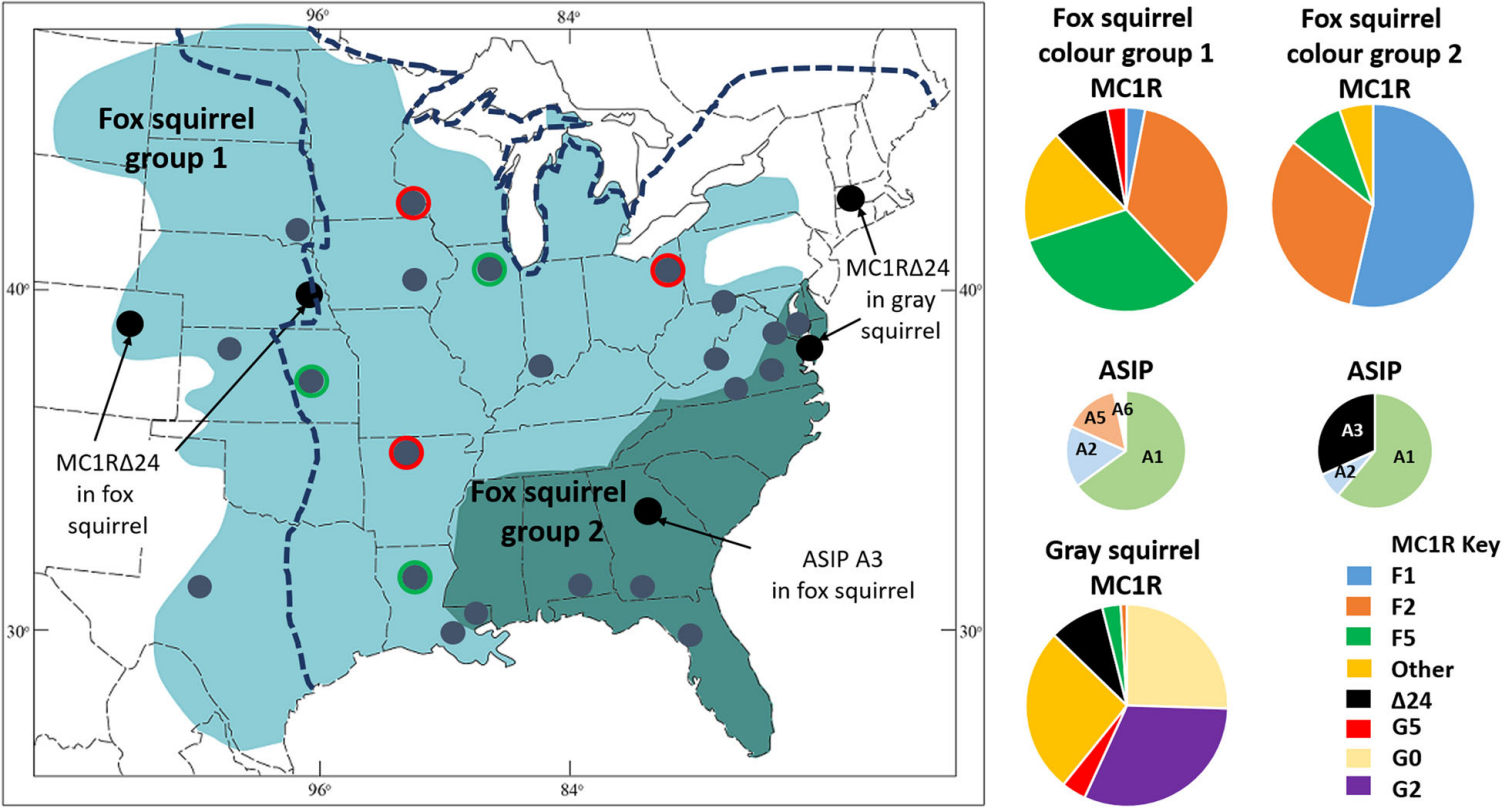
Map of North America showing the native ranges fox squirrels and gray squirrels. Ranges of fox squirrel colour group 1 (orange agouti) are shown in light blue and colour group 2 (gray/tan agouti) in dark green. Gray squirrel range is to the east and south of the heavy dashed line. Spots show the locations of fox squirrel and gray squirrel samples, gray spots = wildtype, and black spots = samples with both wildtype and melanic fox squirrels or gray squirrels. Spots with coloured outlines show locations of fox squirrels with *MC1R* alleles typically from the gray squirrel (red outline) and locations of gray squirrels with *MC1R* alleles typically from the fox squirrel (green outline). Pie charts show *MC1R* haplotype frequencies in the fox squirrel and gray squirrel and *ASIP* genotype frequencies (see Additional file 3) in the fox squirrel

**Fig. 2:**
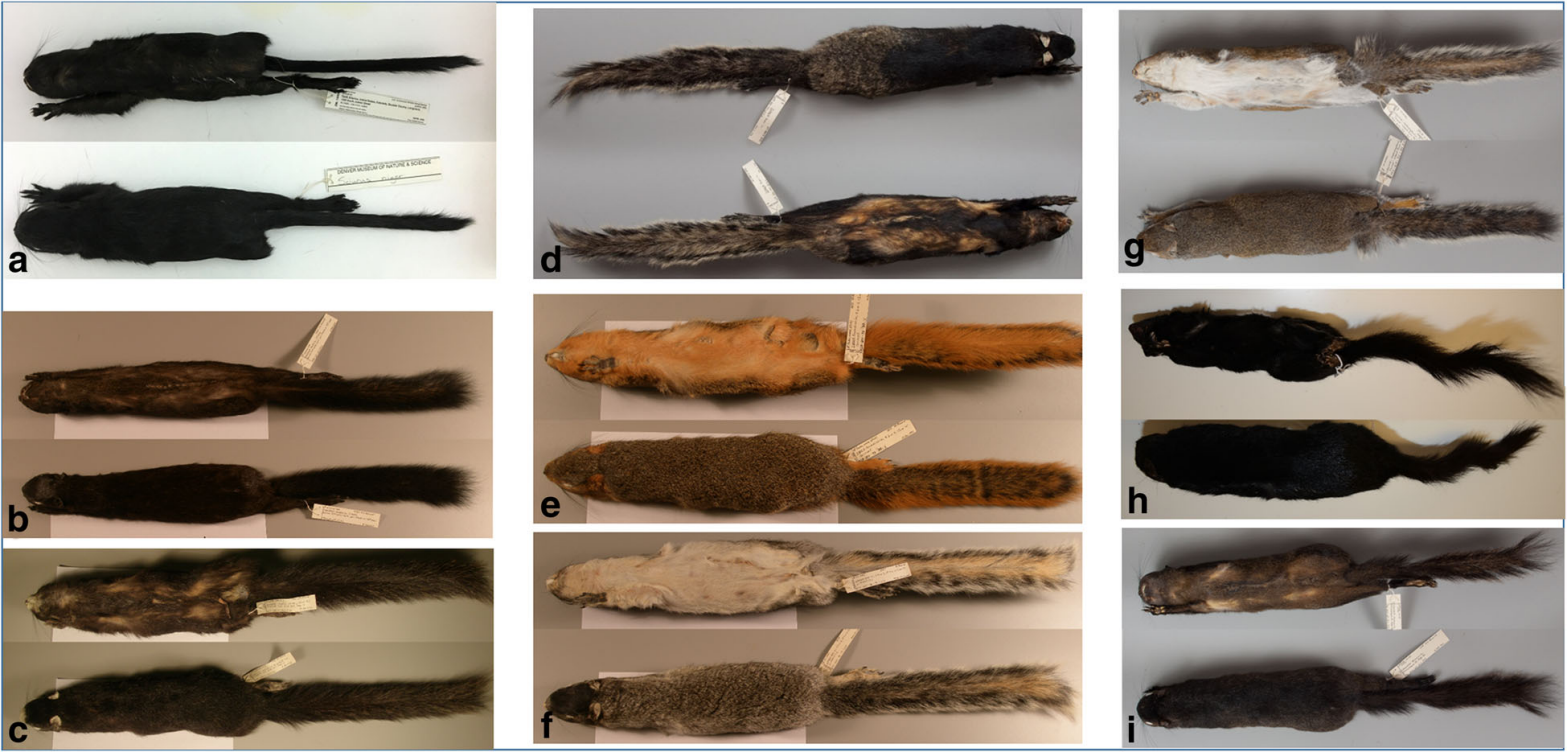
Photos of fox (*Sciurus niger*) and gray (*Sciurus carolinensis*) squirrels. Squirrels are described as jet-black melanic if their entire coat has solid jet-black hairs, as partial melanic if their coat has between 75 and 90% solid jet-black hairs, and as brown-black melanic if their coat is overall darkened, with banding on the hairs. **a**) Colour group 1 (orange agouti) fox squirrel homozygous for the *MC1RΔ24* allele (jet-black melanic). **b**) Colour group 1 fox squirrel heterozygous for the *MC1RΔ24* allele (brown-black melanic). **c**) Colour group 2 (gray/tan agouti) fox squirrel homozygous for the Gly121Cys mutation in *ASIP* (jet-black melanic). **d**) Colour group 2 fox squirrel heterozygous for the Gly121Cys mutation in *ASIP* (partial melanic). **e**) Colour group 1 (orange agouti) wildtype fox squirrel. Wildtype fox squirrels from colour group 1 lack white markings, have an overall orange-red agouti colouration and orange or yellow venters. **f**) Colour group 2 (gray/tan agouti) wildtype fox squirrel. Wildtype fox squirrels from colour group 2 have an overall silver-gray or tan agouti colouration with cream or buff venters and black on the dorsal surface of their heads and often have white markings on their noses, ears, feet, and tails. **g**) Gray squirrel, typical wildtype grizzled phenotype. **h**) Gray squirrel homozygous for the *MC1RΔ24* allele (jet-black melanic). **i**) Gray squirrel heterozygous for the *MC1RΔ24* allele (brown-black melanic)

The fox squirrel and gray squirrel are naturally sympatric over a broad region of eastern North America (Fig. 1) and have similar ecological requirements and life histories [25]. Like many species of wild mammals, individual hairs on the dorsum of these squirrels usually have alternating bands of brown/black (eumelanin) and red/yellow (phaeomelanin) pigments, a pelage condition known as “agouti.” The overall appearance of coat colour for a particular animal depends on the width and placement of the pigment bands along the hair shafts as well as the intensity of the pigments. Coat colour of individuals for some wild mammals, such as the gray squirrel, is relatively uniform over the geographic distribution of the species. Other species, including the fox squirrel, exhibit dramatic patterns of geographic variation in coat colour. There are two distinct colour groups of fox squirrels: animals from most of the range (colour group 1), have an overall orange agouti colouration, whereas animals from the south-eastern coastal plain (colour group 2) are generally silver-gray or tan agouti with black heads and white noses and ears (Fig. 2). The colour group 1 (orange agouti) squirrels generally have intense reddish bands of phaeomelanin in their dorsal hairs; whereas the gray/tan agouti (colour group 2) animals generally have dilute yellowish bands.

Melanism (uniform dark brown or black colouration over the whole body) occurs at low frequency (less than 1%) across most of the range of both fox squirrels and gray squirrels [26, 27]. In this study, squirrels are characterized as jet-black melanic if their entire coat has solid jet-black hairs, as partial melanic if their coat has between 75 and 90% solid jet-black hairs, and as brown-black melanic if their coat is overall darkened, with banding on the hairs (Fig. 2). Melanism in the gray squirrel is much more common in the northern part of the range (more than 75%) [26]; in contrast, melanism in fox squirrels is more common in the southern part of the range, reaching a maximum frequency of 13% [27]. We previously reported that a 24 bp deletion in the *MC1R* is associated with melanism in the gray squirrel, where homozygotes for the mutation are jet-black melanic, heterozygotes are brown-black melanic, and squirrels homozygous or heterozygous for other alleles have a typical grizzled wildtype phenotype (Fig. 2) [28]. The genetic basis of melanism in the fox squirrel has not yet been elucidated.

The aim of this study was to investigate the genetic basis of melanism in the two colour groups of the fox squirrel, using a candidate gene approach. Having found that the same 24 bp deletion in the *MC1R,* which had previously been described in the gray squirrel, was also associated with melanism in the colour group 1 (orange agouti) fox squirrel, the study was expanded to examine the causes of derived allele sharing between the two species. Here we present evidence of multiple genetic origins of adaptive melanism in two species of tree squirrels.

## Results

An allele at the *MC1R* locus with a 24 bp deletion (*MC1R∆24*) is associated with melanism in colour group 1 (orange agouti) fox squirrels from Colorado and Nebraska. One jet-black melanic squirrel was homozygous and seven brown-black melanic squirrels were heterozygous for the *MC1R∆24* allele, whereas all other colour group 1 fox squirrels (*n* = 42) had other alleles (Fisher’s exact test, *P* < 10^− 11^) (Table 1). An *MC1R* allele with the same 24 bp deletion was previously found to be associated with melanism in the gray squirrel [28]. We therefore compared *MC1R* variation in fox squirrels to that from an expanded sample of gray squirrels (*n* = 51) (Table 1 and Additional file 1).

**Table 1:** Samples used in this study. *S*. is *Sciurus*

Species	Sample ID	Colour Group	Location	Phenotype	MC1R genotype	ASIP genotype
*S. niger*	NDM 2380	2	Southern Georgia	Partial melanic	F1/F2	A1/A3
*S. niger*	NDM 2382	2	Southern Georgia	Partial melanic	F1/F2	A1/A3
*S. niger*	NDM 2383	2	Southern Georgia	Partial melanic	F1/F1	A1/A2
*S. niger*	NDM 2386	2	Southern Georgia	Partial melanic	F1/F1	A1/A1
*S. niger*	NDM 2953	2	Southern Georgia	Partial melanic	F1/F1	A2/A3
*S. niger*	NDM 2378	2	Southern Georgia	Wild-type	F1/F2	A1/A1
*S. niger*	NDM 2379	2	Southern Georgia	Wild-type	F1/F2	A1/A1
*S. niger*	NDM 2389	2	Southern Georgia	Wild-type	F1/F1	A1/A1
*S. niger*	NDM 2396	2	Southern Georgia	Wild-type	F1/F6	A1/A1
*S. niger*	NDM 2400	2	Southern Georgia	Wild-type	F1/F2	A1/A1
*S. niger*	NDM 2955	2	Southern Georgia	Wild-type	F1/F2	A1/A1
*S. niger*	NDM 1624	2	Northern Georgia	Jet black	F1/F1	A3/A3
*S. niger*	NDM 1627	2	Northern Georgia	Jet black	F1/F1	A3/A3
*S. niger*	NDM 1526	2	Northern Georgia	Wild-type	F1/F1	A1/A1
*S. niger*	NDM 1539	2	Northern Georgia	Wild-type	F1/F2	A1/A1
*S. niger*	NDM 1544	2	Northern Georgia	Wild-type	F1/F2	A1/A1
*S. niger*	NDM 1550	2	Northern Georgia	Wild-type	F1/F2	A1/A1
*S. niger*	NDM 1551	2	Northern Georgia	Wild-type	F2/F2	A1/A2
*S. niger*	NDM 1553	2	Northern Georgia	Wild-type	F1/F1	A1/A1
*S. niger*	NDM 1575	2	Northern Georgia	Wild-type	F1/F2	A1/A2
*S. niger*	NDM 1589	2	Northern Georgia	Wild-type	F1/F2	A1/A2
*S. niger*	NDM 1519	2	Northern Georgia	Partial melanic	F1/F1	A1/A3
*S. niger*	NDM 1585	2	Northern Georgia	Partial melanic	F1/F1	A2/A3
*S. niger*	NDM 1616	2	Northern Georgia	Partial melanic	F1/F1	A1/A3
*S. niger*	NDM 1631	2	Northern Georgia	Partial melanic	F1/F2	A1/A3
*S. niger*	NDM 1534	2	Northern Georgia	Jet black	F1/F1	A3/A3
*S. niger*	NDM 1540	2	Northern Georgia	Jet black	F1/F2	A3/A3
*S. niger*	NDM 1595	2	Northern Georgia	Jet black	F1/F1	A3/A3
*S. niger*	NDM 1609	2	Northern Georgia	Jet black	F1/F1	A3/A3
*S. niger*	NDM 1621	2	Northern Georgia	Jet black	F1/F2	A3/A3
*S. niger*	NDM 1641	2	Northern Georgia	Jet black	F1/F1	A3/A3
*S. niger*	NDM 1545	2	Northern Georgia	Wild-type	F1/F2	A1/A1
*S. niger*	NDM 1642	2	Northern Georgia	Jet black	F1/F2	A3/A3
*S. niger*	NDM 1535	2	Northern Georgia	Wild-type	F1/F3	A1/A1
*S. niger*	NDM 1563	2	Northern Georgia	Wild-type	F1/F1	
*S. niger*	NDM 1570	2	Northern Georgia	Wild-type	F1/F2	A1/A1
*S. niger*	NDM 1607	2	Northern Georgia	Wild-type	F1/F1	A1/A1
*S. niger*	NDM 1612	2	Northern Georgia	Wild-type	F1/F2	A1/A3
*S. niger*	NDM 1614	2	Northern Georgia	Wild-type	F1/F1	A1/A1
*S. niger*	NDM 1617	2	Northern Georgia	Wild-type	F2/F3	A1/A1
*S. niger*	NDM 1622	2	Northern Georgia	Wild-type	F1/F3	A1/A2
*S. niger*	NDM 1629	2	Northern Georgia	Wild-type	F2/F2	A1/A1
*S. niger*	NDM 2232	1	Iowa	Wild-type	F4/F4	A1/A1
*S. niger*	NDM 2233	1	Iowa	Wild-type	F4/F2	A1/A1
*S. niger*	NDM 2229	1	Kansas	Wild-type	F2/F3	A1/A1
*S. niger*	NDM 1711	1	Maryland	Wild-type	F2/F5	A1/A1
*S. niger*	NDM 4304	1	Nebraska	Brown black	Δ24/F2	A1/A1
*S. niger*	NDM 4305	1	Nebraska	Brown black	Δ24/F2	A1/A1
*S. niger*	NDM 3612	1	South Dakota	Wild-type	F2/F5	A1/A1
*S. niger*	NDM 3613	1	South Dakota	Wild-type	F2/F4	A1/A1
*S. niger*	NDM 3615	1	South Dakota	Wild-type	F2/F5	A1/A1
*S. niger*	NDM 1780	1	Virginia	Wild-type	F2/F2	A2/A2
*S. niger*	NDM 2196	1	Virginia	Wild-type	F2/F2	A2/A2
*S. niger*	NDM 1782	1	Virginia	Wild-type	F2/F5	A1/A1
*S. niger*	NDM 1783	1	Virginia	Wild-type	F2/F2	A1/A1
*S. niger*	NDM 2193	1	Virginia	Wild-type	F2/F5	A1/A2
*S. niger*	NDM 2194	1	Virginia	Wild-type	F2/F2	A1/A1
*S. niger*	NDM 1682	1	Virginia	Wild-type	F3/F2	A2/A2
*S. niger*	NDM 1790	1	Virginia	Wild-type	F1/F2	A1/A1
*S. niger*	NDM 2211	1	Virginia	Wild-type	F2/F2	A1/A1
*S. niger*	NDM 1426	2	Alabama	Wild-type	F1/F1	
*S. niger*	NDM 1460	2	Maryland	Wild-type	F5/F5	
*S. niger*	NDM 1464	2	Maryland	Wild-type	F2/F5	
*S. niger*	NDM 1469	2	Maryland	Wild-type	F2/F5	
*S. niger*	NDM 1483	2	Maryland	Wild-type	F2/F2	
*S. niger*	NDM 1485	2	Maryland	Wild-type	F2/F7	
*S. niger*	NDM 1486	2	Maryland	Wild-type	F2/F5	
*S. niger*	NDM 1490	2	Maryland	Wild-type	F2/F5	
*S. niger*	NDM 1744	1	Indiana	Wild-type	F5/F5	
*S. niger*	NDM 1753	1	Indiana	Wild-type	F5/F5	
*S. niger*	NDM 1762	1	Illinois	Wild-type	F5/F5	
*S. niger*	NDM 1763	1	Illinois	Wild-type	F4/F4	
*S. niger*	NDM 2214	1	Kansas	Wild-type	F4/F2	
*S. niger*	NDM 2215	1	Kansas	Wild-type	F2/F5	
*S. niger*	NDM 2216	1	Kansas	Wild-type	F4/F4	
*S. niger*	NDM 2217	1	Kansas	Wild-type	F2/F5	
*S. niger*	NDM 2218	1	Kansas	Wild-type	F5/F5	
*S. niger*	NDM 2219	1	Kansas	Wild-type	F2/F5	
*S. niger*	NDM 2220	1	Kansas	Wild-type	F4/F5	
*S. niger*	NDM 2221	1	Kansas	Wild-type	F4/F5	
*S. niger*	NDM 2222	1	Kansas	Wild-type	F2/F5	
*S. niger*	NDM 2228	1	Kansas	Wild-type	F2/F2	
*S. niger*	NDM 2243	1	Texas	Wild-type	F1/F1	
*S. niger*	NDM 2244	1	Texas	Wild-type	F5/F10	
*S. niger*	NDM 2245	1	Texas	Wild-type	F11/F5	
*S. niger*	NDM 2246	1	Texas	Wild-type	F5/F5	
*S. niger*	NDM 2248	1	Texas	Wild-type	F11/F5	
*S. niger*	NDM 2249	1	Texas	Wild-type	F5/F5	
*S. niger*	NDM 3331	2	Virginia	Wild-type	F5/F5	
*S. niger*	NDM 3354	2	Virginia	Wild-type	F2/F5	
*S. niger*	NDM 3364	2	Virginia	Wild-type	F5/F7	
*S. niger*	NDM 3660	2	Virginia	Wild-type	F2/F2	
*S. niger*	NDM 3661	2	Virginia	Wild-type	F2/F2	
*S. niger*	VT 1076	1	Minnesota	Wild-type	G5/F5	
*S. niger*	NDM 1052	1	Louisiana	Wild-type	F5/F5	
*S. niger*	NDM 1053	1	Louisiana	Wild-type	F2/F3	
*S. niger*	NDM 1277	1	Louisiana	Wild-type	F5/F9	
*S. niger*	NDM 1322	2	Louisiana	Wild-type	F1/F2	
*S. niger*	DMNS 14926	1	Colorado	Brown black	Δ24/F2	A5/A2
*S. niger*	DMNS 14406	1	Colorado	Jet black	Δ24/Δ24	A1/A1
*S. niger*	DMNS 14268	1	Colorado	Brown black	Δ24/F2	A6/A1
*S. niger*	DMNS 14170	1	Colorado	Brown black	Δ24/F2	A1/A1
*S. niger*	DMNS 17421	1	Colorado	Brown black	Δ24/F2	A6/A1
*S. niger*	DMNS 17359	1	Colorado	Brown black	Δ24/F2	A1/A1
*S. niger*	OMNH 46253	1	Ohio	Melanic^a^	G5/F5	A5/A5
*S. niger*	OMNH 46667	1	Arkansas	Melanic^a^	G5/F5	A5/A5
*S. carolinensis*	AG1		Washington DC	Wild-type	G0/G9	
*S. carolinensis*	AG2		British Columbia	Wild-type	G2/G2	
*S. carolinensis*	AG3		British Columbia	Wild-type	G2/G2	
*S. carolinensis*	AG4		British Columbia	Wild-type	G2/G2	
*S. carolinensis*	AB1		Massachusetts	Jet black	Δ24/Δ24	
*S. carolinensis*	AB2		British Columbia	Brown black	G2/Δ24	
*S. carolinensis*	AB3		British Columbia	Brown black	G2/Δ24	
*S. carolinensis*	AB4		British Columbia	Brown black	G2/Δ24	
*S. carolinensis*	AB5		British Columbia	Brown black	G1/Δ24	
*S. carolinensis*	AB6		British Columbia	Brown black	G1/Δ24	
*S. carolinensis*	NDM 1497		Virginia	Brown black	G2/Δ24	
*S. carolinensis*	NDM 1498		Virginia	Brown black	G1/Δ24	
*S. carolinensis*	NDM 2757		Virginia	Wild-type	G1/G2	
*S. carolinensis*	NDM 2819		Virginia	Wild-type	G2/G7	
*S. carolinensis*	NDM 3818		Virginia	Wild-type	G1/G2	
*S. carolinensis*	NDM 1420		Alabama	Wild-type	G0/G0	
*S. carolinensis*	NDM 1421		Alabama	Wild-type	G0/G0	
*S. carolinensis*	NDM 1422		Alabama	Wild-type	G2/G7	
*S. carolinensis*	NDM 1423		Alabama	Wild-type	G0/G2	
*S. carolinensis*	NDM 1525		Georgia	Wild-type	G2/G0	
*S. carolinensis*	NDM 1591		Georgia	Wild-type	G2/G4	
*S. carolinensis*	NDM 1592		Georgia	Wild-type	G2/G0	
*S. carolinensis*	NDM 1593		Georgia	Wild-type	G0/G4	
*S. carolinensis*	NDM 1633		Georgia	Wild-type	G9/G7	
*S. carolinensis*	NDM 1638		Georgia	Wild-type	G0/G0	
*S. carolinensis*	NDM 1721		Maryland	Wild-type	G2/G7	
*S. carolinensis*	NDM 1722		Maryland	Wild-type	G2/G4	
*S. carolinensis*	NDM 1723		Maryland	Wild-type	G2/G4	
*S. carolinensis*	NDM 1730		Indiana	Wild-type	G4/G4	
*S. carolinensis*	NDM 1731		Indiana	Wild-type	G2/G4	
*S. carolinensis*	NDM 1799		Virginia	Wild-type	G2/G0	
*S. carolinensis*	NDM 1802		Virginia	Wild-type	G2/G0	
*S. carolinensis*	NDM 1803		Virginia	Wild-type	G0/G0	
*S. carolinensis*	NDM 1813		Virginia	Wild-type	G0/G0	
*S. carolinensis*	NDM 1817		Virginia	Wild-type	G0/G2	
*S. carolinensis*	NDM 2258		Virginia	Wild-type	G0/G8	
*S. carolinensis*	NDM 2261		Virginia	Wild-type	G2/G11	
*S. carolinensis*	NDM 2330		Maryland	Wild-type	G0/G2	
*S. carolinensis*	NDM 2331		Maryland	Wild-type	G0/G2	
*S. carolinensis*	NDM 1760		Illinois	Wild-type	G5/F5	
*S. carolinensis*	VT 0985		Kansas	Wild-type	G5/F5	
*S. carolinensis*	VT 0992		Florida	Wild-type	G0/G7	
*S. carolinensis*	VT 0994		Florida	Wild-type	G2/G4	
*S. carolinensis*	NDM 0485		Louisiana	Wild-type	G0/G10	
*S. carolinensis*	NDM 1007		Louisiana	Wild-type	G1/G9	
*S. carolinensis*	NDM 1008		Louisiana	Wild-type	G2/G9	
*S. carolinensis*	NDM 1120		Louisiana	Wild-type	F2/G5	
*S. carolinensis*	NDM 1121		Louisiana	Wild-type	G5/F5	
*S. carolinensis*	NDM 1272		Louisiana	Wild-type	G0/G2	
*S. carolinensis*	NDM 1279		Louisiana	Wild-type	G0/G2	
*S. carolinensis*	NDM 1313		Louisiana	Wild-type	G0/G7	

Melanic^a^ indicates two brown-black melanic fox squirrels where the underlying genetics is unknown

**Fig. 3:**
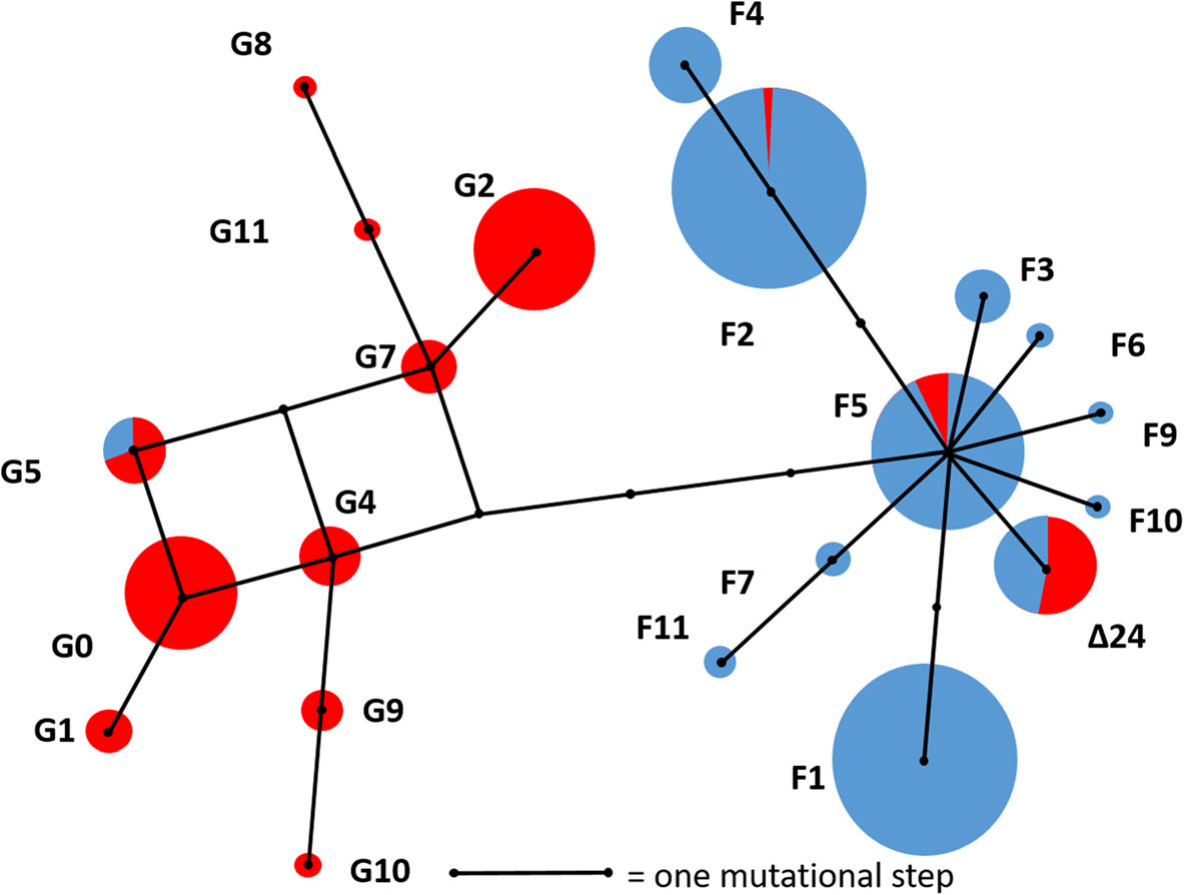
Median joining *MC1R* haplotype network for the gray squirrel and fox squirrel. Each circle represents an allele and each line represents one or more substitutions or a deletion. Alleles from fox squirrel are blue, alleles from gray squirrel are red. Area of circles is proportional to the numbers of alleles sampled

**Fig. 4:**
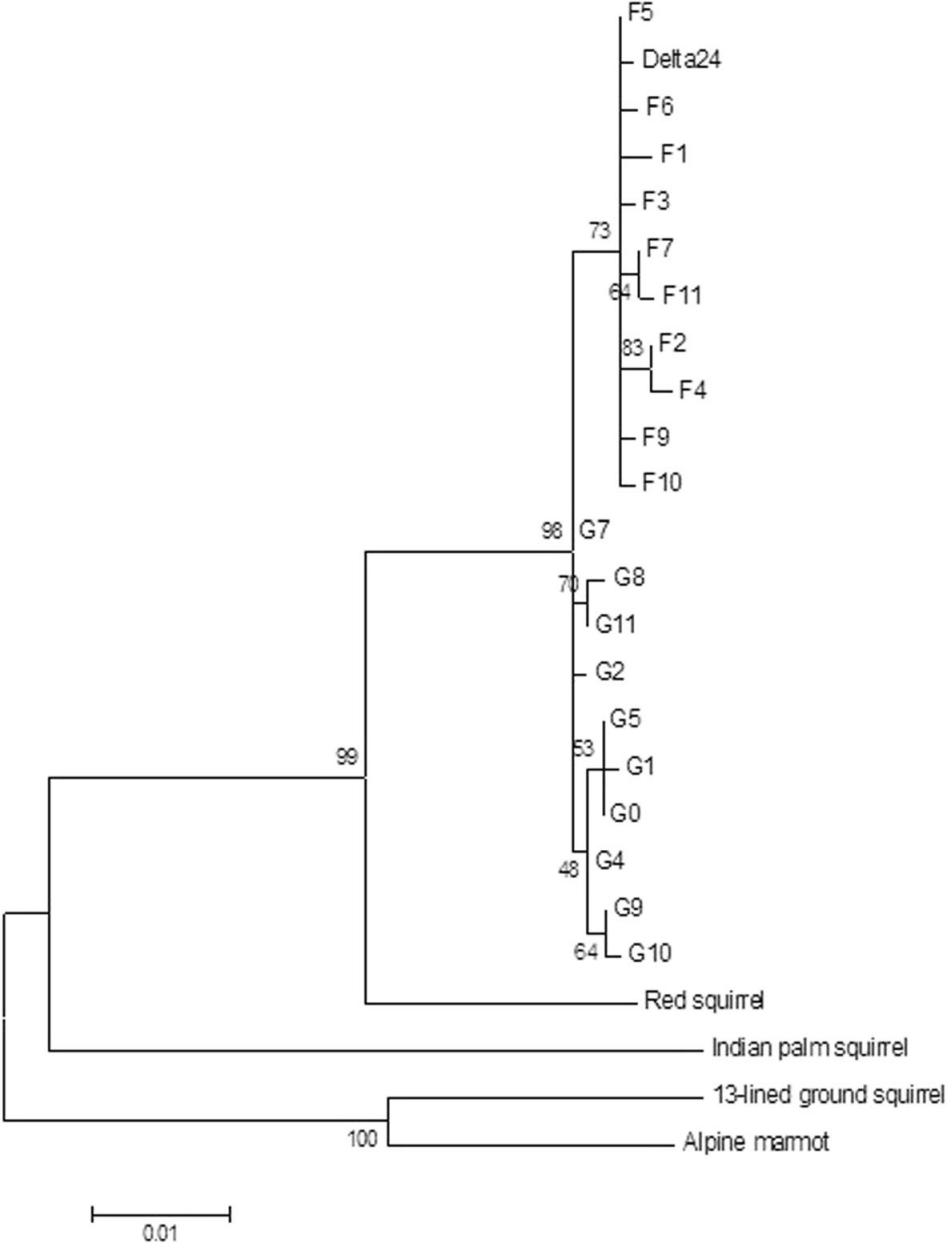
Phylogenetic reconstruction of *MC1R* haplotypes in squirrels. Maximum likelihood reconstruction with bootstrap support values on branches, and branch lengths proportional to sequence evolution. *MC1R* haplotypes for fox squirrels and gray squirrels are included separately (see Fig. 3)

Remarkably, all *MC1R* haplotypes containing the 24 bp deletion are identical in the two species (allele counts 9 in fox squirrels and 9 in gray squirrels), and this includes gray squirrel populations introduced to British Columbia, Canada in the early 1900’s and gray squirrels introduced to Britain from North America in the late 1800’s [29]. On a haplotype network, the *MC1R∆24* allele is nested within the alleles from fox squirrels, a minimum of 4 mutational steps away from all other common alleles in gray squirrels (Fig. 3, Additional file 1). Most other alleles form species-specific clusters, but rare alleles in both species are also shared. Bayesian modelling of gene flow with a two population model (Table 2, Additional file 2) shows consistent estimation of a low degree of bi-directional gene flow between the two species, with estimates of gene flow in both directions significantly greater than zero (at *p* < 0.01) in all runs (e.g. run 1: gray to fox, LLR (log likelihood ratio) = 13.81, *p* < 0.01, fox to gray, LLR = 13.51, *p* < 0.01). Phylogenetic reconstructions using maximum likelihood show that the fox squirrel *MC1R* alleles together with the *MC1R∆24* allele form a monophyletic clade that has 73% bootstrap support (Fig. 4).

**Table 2:** Parameter estimates from three iMa2 runs, with 95% upper and lower posterior density bounds

Run	Fox Ɵ	Gray Ɵ	Gene flow (2N_e_m)	Gene flow (2N_e_m)
			Gray to fox	Fox to gray
1	1.74 (0.96–3.64)	1.96 (1.04–4.41)	0.11 (0.029–0.47)	0.31 (0.077–0.49)
2	1.74 (0.96–3.64)	1.96 (1.04–4.36)	0.11 (0.029–0.47)	0.31 (0.077–0.49)
3	1.76 (0.94–3.64)	1.96 (1.04–4.41)	0.11 (0.029–0.47)	0.31 (0.077–0.49)

**Fig. 5:**
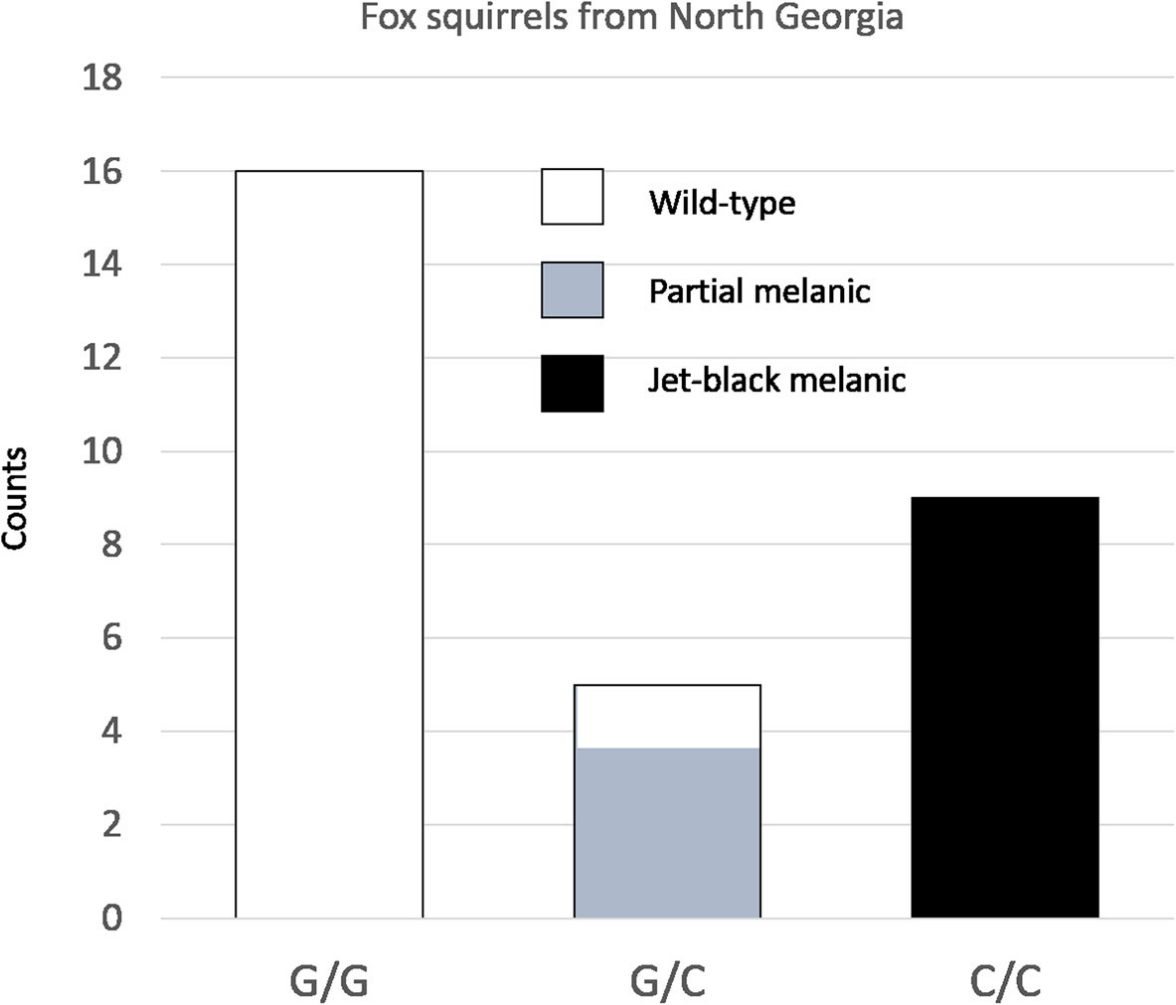
Association between *ASIP* genotypes and phenotypes of fox squirrels from northern Georgia. Counts of the three phenotypes are given for the three genotypes defined by variation at nucleotide position 361 (Gly121Cys mutation present on haplotype A3)

There was no association between *MC1R* and melanism in colour group 2 (gray/tan agouti) fox squirrels (Table 1). Here there was an association between melanism and variation in *ASIP*: all nine jet-black melanic individuals, sampled from a single population in northern Georgia, were homozygous for a single bp substitution (G361T) leading to a Gly121Cys mutation, whereas all other colour group 2 individuals (*n* = 32) were heterozygous or homozygous for other alleles (Fisher’s exact test: *P* < 10^− 10^) (Table 1 and Additional file 3). There was also a strong tendency for individuals with intermediate, partial melanic colouration to be heterozygous for the Gly121Cys mutation, with a significant association across all colour group 2 squirrels (Fisher’s exact test: *P* < 10^− 5^). The associations between *ASIP* genotype and melanism are shown for the northern Georgia population in Fig. 5. The G361T substitution is unique to *ASIP* haplotype A3 (Additional file 3). A C253G substitution, causing an Arg85Gly mutation, which is also present in haplotype A3, is not associated with melanism. This is shown by haplotype A2, which has the C253G but not the G361T substitution: haplotype A2 is never seen in a jet-black squirrel, and almost all A1/A2 heterozygotes are wild-type and both A2/A3 heterozygotes are partial melanic (Table 1). Two melanic colour group 1 (orange agouti) fox squirrels, from Ohio and Arkansas, had no evidence for mutations in coding regions of the *MC1R* or *ASIP* associated with phenotype, suggesting a further genetic mechanism for melanism.

## Discussion

We present strong evidence for identifying the loci underlying convergent evolution of melanism in two populations of fox squirrels. Melanism in colour group 1 (orange agouti) fox squirrels from Colorado and Nebraska is associated with the *MC1R∆24* allele identical to that found in the gray squirrel. In contrast, colour group 2 (gray/tan agouti) fox squirrels do not show an association between *MC1R* and melanism (which was the basis of our previous report of a lack of association) [29], and the *MC1R∆24* allele is absent in this population. The 24 bp deletion falls at a mutational hotspot on the boundary of the second and third transmembrane domains in the MC1R receptor where a number of other species have mutations associated with melanism, for example, the bananaquit which has a E92K mutation causing the receptor to be constitutively active [30]. Functional studies on the MC1R∆24 protein in the gray squirrel confirmed that it plausibly causes melanism – it showed high basal activity as well as responding to ASIP as an agonist in comparison to the usual inverse agonist activity of ASIP [31]. There are further examples of deletions in this part of the receptor leading to a darkened phenotype in wild populations including Eleonora’s falcon, the jaguar, the jaguarundi and the golden-headed lion tamarin [20, 32, 33].

**Fig. 6:**
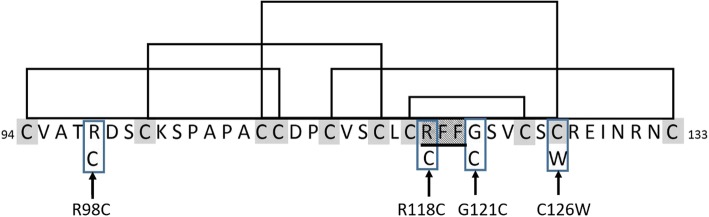
Cysteine-rich domain of the agouti signalling protein. Sequence of the cysteine-rich domain of the fox squirrel (all numbering follows squirrel ASIP sequence). Disulphide bridges are shown as black lines connecting cysteines which are shaded. The bridges are predicted from those of mouse and human ASIP [36]. Mutations in the fox squirrel (Gly121Cys), dog and alpaca (Arg98Cys), [37, 38], pampas cat (Arg118Cys) [21] and Asian golden cat (Cys126Trp), [24] are boxed and highlighted with arrows. The highly conserved RFF sequence is underlined and shaded

We found that jet-black melanism and partial melanism in colour group 2 (gray/tan agouti) fox squirrels, are associated with a non-synonymous single nucleotide polymorphism in the *ASIP* locus, with all jet-black individuals being homozygous for the derived allele, and most partial melanic squirrels being heterozygous. Jet-black melanic, partial melanic, and wildtype squirrels were all sampled from the same location in northern Georgia, indicating frequent interbreeding in relation to colour phenotype. Hence population structure cannot explain this association, and this is further supported by the absence of an association between colouration and *MC1R* genotype, and the overall low frequency of jet-black individuals in group 2 fox squirrels (maximum 13%). Interestingly, since there is a strong trend for heterozygous individuals to have an intermediate colour phenotype, our results are most consistent with a pattern of partial dominance at *ASIP*, which is unusual [34]. The Gly121Cys mutation falls in the highly conserved cysteine-rich domain of the ASIP protein, which is thought to form a highly ordered structure stabilised by five disulphide bridges that create an inhibitor cysteine-knot motif (Fig. 6 and Additional file 4) [35, 36]. The position of 10 cysteine residues in this region is highly conserved across mammalian orders, and in the four cases where changes in the number of cysteine residues have been reported, they are associated with melanism: German Shepherd dogs and alpacas (Arg96Cys) [37, 38], the pampas cat (Arg120Cys) [21] and the Asian golden cat (Cys128Trp) [24]. Taken together this strongly suggests that the Gly121Cys mutation in ASIP is causative for melanism in colour group 2 (gray/tan agouti) fox squirrels, although functional studies will be needed to confirm this. Our findings indicate that melanism has evolved at least twice in fox squirrels: jet-black melanic phenotypes in different parts of the species’ range are the result of mutations at two different loci, *MC1R* and *ASIP.* These two loci are also associated with intraspecific events of parallel melanism in a Solomon Island flycatcher [22] and in rock pocket mice [39, 40]. These results in tree squirrels add to the extensive evidence that *MC1R* and *ASIP* represent functionally equivalent “adaptive hotspots” for melanism in vertebrates [18].

It is intriguing that the identical *MC1R∆24* haplotype is associated with melanism in both fox squirrel and gray squirrel species, and there are three possible explanations for how this has occurred. First, the allele could have arisen in the common ancestor of both species, and been retained by balancing selection. This is unlikely since deep divergences between clusters of haplotypes with and without the deletion would be expected. Second, the mutation could have arisen independently in both species, but this is also unlikely as the haplotypes are identical. Therefore the most likely explanation is that the *MC1R∆24* allele arose in one species and subsequently introgressed to the other species. Given the close association of the *MC1R∆24* allele with common fox squirrel haplotypes, and the support for monophyly of fox squirrel haplotypes including the *MC1R∆24* allele, introgression from the fox squirrel into the gray squirrel is by far the most likely scenario, but we cannot completely rule out the possibility of introgression in the other direction. The plausibility of introgression between these two sympatric species is supported by the Bayesian modelling results, although caution is needed since the results are based on a single locus, and a couple of parameters had broad posterior curves. It is notable that these squirrels have been observed in mixed-species mating chases, with male fox squirrels pursuing female gray squirrels [41].

Interspecies mating is likely to be an important source of adaptive genetic variation. It has been noted that introgressive hybridisation has the largest evolutionary impact if two species have some morphological differences but are still closely related enough to recognise the other species as potential mates and be reproductively compatible and before the point reached when genetic incompatibilities incur severe fitness costs [8]. In such cases introgression can provide genetic variants at a higher frequency than de novo mutation, thus accelerating the evolutionary process. Unlike novel mutations, adaptive introgression has the advantage of involving alleles that have already been tested by natural selection, and so where adaptive alleles are “available” in closely related species, they are likely to make an important contribution. Furthermore, dominant alleles are more likely to become established by introgression than recessive alleles, for example in F_1_ individuals among the parental species where the beneficial effects of the dominant introgressed allele may counteract outbreeding depression at other loci. This is precisely the pattern found here, where dominant melanic *MC1R* alleles have introgressed rather than recessive *ASIP* melanic alleles. It would be interesting to perform population genomic analyses on these populations to investigate the possibility of adaptive introgression of *MC1R* further.

In some cases hybridization can expand the ecological niche of a population by increasing physiological tolerances beyond the range of either of the parental species [8, 42]. This is particularly relevant where there is a new ecological challenge which may occur at the periphery of a population’s range, such as a cold climate [4, 8]. For example, we suggest that the high frequency of melanic gray squirrels (with the *MC1R∆24* allele) in the northern parts of the species’ range, which was first noted in the 1740s by early European explorers of North America [43], might be explained by a thermogenic advantage in cold climates [44]. We also suggest that this high frequency of melanism may have contributed to the pre-historic expansion of the gray squirrel’s range (during the past 11,000 years following the Wisconsinan glaciation) further north into eastern Canada. Melanism associated with the *MC1R∆24* allele may also confer thermal advantage to colour group 1 (orange agouti) fox squirrels that inhabit regions with extremely cold, harsh winters, such as Nebraska and Colorado [45]. On the other hand, melanism probably does not confer thermal advantage to colour group 1 (orange agouti) fox squirrels in the southern part of the range (lower Mississippi River drainage), because those animals rarely (if ever) experience temperatures as low as those consistently recorded in Nebraska and Colorado. Thus, the adaptive advantage for melanism appears to differ between gray squirrels and some fox squirrels, and factors responsible for melanism may differ among populations of the fox squirrel.

In addition to providing thermal advantage, melanism often functions to camouflage animals from predators [12, 13]. To account for the higher frequency of melanism in the southern part of the fox squirrel’s range, Kiltie [27] posed the hypothesis that melanism increases camouflage of colour group 2 (gray/tan agouti) fox squirrels from predators (hawks) in areas frequently burned by wildfires, such as those on the south-eastern coastal plain. He subsequently conducted a series of experiments to test concealment of all phenotypes of both fox squirrel colour groups [46, 47]. He tested dynamic crypsis by presenting captive red-tailed hawks (*Buteo jamaicensis*) with moving models of melanic and wildtype phenotypes against different backgrounds (including burned and unburned tree bark) [46]. He also tested static crypsis by analysing digitized photographs to determine how well museum specimens of melanic and wildtype phenotypes matched different background types (including burned and unburned tree bark) [45, 46]. Kiltie’s experiments yielded complex results: for both colour groups, he concluded that melanism may confer concealment from hawks when fox squirrels are in motion, but wildtype colouration may be better camouflage when the animals are not moving [46, 47]. Clearly much work remains to be done to elucidate the selective pressures on melanism in tree squirrels.

## Conclusions

We conclude that the presence of the *MC1R∆24* allele and melanism in gray squirrels is likely due to introgression from colour group 1 (orange agouti) fox squirrels. We further conclude that melanism in colour group 2 (gray/tan agouti) fox squirrels is associated with a Gly121Cys mutation in ASIP. Finally, convergent melanism in these two species of tree squirrels has evolved by at least two and likely three different evolutionary routes – *MC1R* mutation, *ASIP* mutation, and probably introgression of *MC1R* mutation.

## Methods

### Sampling

We used tissues and DNA samples originally collected as part of previous genetic studies of these species [28, 29, 48, 49], and we also obtained tissues from museum specimens housed at Louisiana State University Museum of Zoology, Sam Noble Oklahoma Museum of Natural History, and Denver Museum of Natural Sciences. (Note that all samples were originally collected following methods which met the guidelines of the American Society of Mammalogists for the use of mammals in research). In total we used tissue samples from 106 fox squirrels and 51 Gray squirrels from 28 locations across their ranges (see Figs. 1 and 2, Table 1). Permission was granted to import squirrel tissue to the United Kingdom from the United States of America by the Department for Environment, Food and Rural Affairs: authorisation number IMP/GEN/2014/06.

### Genotyping

We extracted genomic DNA from tissue (muscle or skin) using a DNeasy Blood and Tissue Kit (Qiagen). We amplified the full coding sequence of the *MC1R* gene by polymerase chain reaction (PCR) using MC1R13li forward primer (TTT TCT GAG GAC AGA TCA ATG) and MC1Rer10 reverse primer (AGA ATC CAC CCT CCC TGC TC). Approximately 25 ng template DNA was used in a total volume of 25 μl with 2 × MyTaq Red Mix (Bioline) and 0.4 μM primers under the following conditions: initial denaturation 94 °C for 2 min, 30 cycles of 94 °C for 30 s, 63 °C for 30 s, and 72 °C for 1 min, followed by a final extension at 72 °C for 5 min. We amplified all three coding exons of the *ASIP* gene using the following primers: exon 2: ASP2_13LF1 forward primer (5′-AGT ACT CCG CCC TCT GGA TA-3′) and ASP3sqR11 reverse primer (5′-TCT GCT TCT TTT CTG CTG AT-3′), exon 3: ASPsqintron2to3F1 (5′-CCC TCT GCT CCT TCC ATT TT-3′) and ASPintron3to4R1 (5′-AAT GAG AAC TCC CAG GCC TAC-3'), exon 4: ASP3to4sqF11 (5′-ATG GAC AGC TCC CGC ATT T-3′) and ASP4sqrex30 (5′-AGG AAG CTT TGA GTG GAC GA-3′). PCR conditions were as for the *MC1R* but with annealing temperatures as follows: exon 2, 64 °C, exon 3, 55 °C and exon 4, 62 °C. All PCR products were Sanger sequenced on both strands at the Department of Biochemistry, University of Cambridge.

### Haplotype reconstruction

We used PHASE 2.1 [50] to reconstruct haplotypes of the *MC1R*, and Network [51] to generate median joining networks. All sequences have been deposited on Genbank with the following accession numbers: G0: EU604831, G1: KF188573, G2: KF188574, G4 KF188576, G5: KF188577, G7: MH043118, G8: MH043120, G9: MH043119, G10: MH043117, G11: MH043121, MC1RΔ24: EU604830, F1: KF052119, F2: KF052120, F3: MH043124, F4: MH043129, F5: MH043122, F6: MH043125, F7: MH043123, F9: MH043127, F10: MH043128, F11: MH043126, A1: KU724106, A2: KU724107, A3: KU724108, A4: MK780223, A5: MK780221, A6: MK780222.

### Phylogenetic analysis

We used *MC1R* sequences of other sciurid rodents from Genbank as outgroups (red squirrel, *Sciurus vulgaris*, Accession KF188571; thirteen-lined ground squirrel, *Ictidomys tridecemlineatus* XM 005342828; Indian palm squirrel, *Funambulus palmarum*, KC14989; alpine marmot, *Marmota marmota,* XM 015495923). We used JModelTestv2.0 [52] to estimate the optimal substitution model according to Bayesian Information Criterion (BIC); the optimal model was HKY + G. Maximum likelihood reconstructions were performed in MEGA v6.0 [53], with branch support assessed using 500 bootstrap samples.

### Gene flow analysis

Bayesian modelling was used to assess gene flow between fox squirrels and gray squirrels. We used iMa2 [54] to estimate gene flow at *MC1R* between fox (*n* = 106) and gray (*n* = 39) squirrels in eastern USA in a two population model, ignoring the delta24 deletion. We conducted short preliminary runs to determine upper bounds for the demographic parameters and appropriate heating parameters. Then we conducted three independent runs with a different random number seed, for 10^6^ MCMC steps and a burn-in period of 10^5^ steps. We used 40 chains with heating parameters -ha 0.975, −hb 0.75. Convergence was assessed by the concordance of parameter estimates, acceptable chain mixing and autocorrelations.

## Additional files

Additional file 1: *“MC1R* haplotypes from fox squirrels (F-) and gray squirrels (G-) with the *MC1RΔ24* haplotype as reference.” Table showing MC1R haplotypes. (DOCX 31 kb)

Additional file 2: “Marginal posterior density distributions of parameters from iMa2 analyses.” Graphs showing marginal posterior density distributions of parameters from iMa2 analyses. (PPTX 51 kb)

Additional file 3: “*ASIP* haplotypes (coding sequence) from the fox squirrel (A1 – A3, A5, A6) and gray squirrel (A4) with the most common haplotype as reference.” A table showing ASIP haplotypes. (DOCX 12 kb)

Additional file 4: “Alignment of ASIP protein sequences from diverse rodents and other mammalian taxa.” A table showing an alignment of ASIP protein sequences from diverse rodents and other mammalian taxa. (DOCX 14 kb)

## Data Availability

All DNA sequence data is available through the publically available Genbank database.

## Abbreviations

ASIP: Agouti signalling protein
MC1R: Melanocortin-1-receptor gene
*MC1RΔ24*: Melanocortin-1-receptor with a 24 base pair deletion
