# Supplementary material for: Multiple origins of melanism in two species of North American tree squirrel (Sciurus)
Source: BMC Evol Biol. 2019 Jul 11;19:140. doi: 10.1186/s12862-019-1471-7 (PMC6625063; doi:10.1186/s12862-019-1471-7)
Supplement: Supplementary file 1 — “MC1R haplotypes from fox squirrels (F-) and gray squirrels (G-) with the MC1RΔ24 haplotype as reference.” Table showing MC1R haplotypes. (DOCX 31 kb) [file 12862_2019_1471_MOESM1_ESM.docx]

Additional file 1. *MC1R* haplotypes from fox squirrels (F-) and gray squirrels (G-) with the *MC1RΔ24* haplotype as reference. Bold underlined numbers refer to non-synonymous substitutions. Dashes indicate consensus and Δ indicates deleted base pairs. Dif. Δ24 is the number of mutational steps between the *MC1RΔ24* haplotype and each allele.

| Base pair/  Allele | 43 | 60 | 84 | **88** | 105 | 156 | 180 | Del  259-282 | **314** | 324 | 438 | 456 | 462 | 474 | 486 | **487** | 501 | 546 | **568** | 591 | 678 | **698** | 783 | Dif. Δ24 |
| --- | --- | --- | --- | --- | --- | --- | --- | --- | --- | --- | --- | --- | --- | --- | --- | --- | --- | --- | --- | --- | --- | --- | --- | --- |
| Δ24 | C | G | C | C | A | G | C | Δ | C | C | G | C | G | G | C | G | G | C | G | A | C | G | C | - |
| F5 | - | - | - | - | - | - | - | A-C | - | - | - | - | - | - | - | - | - | - | - | - | - | - | - | 1 |
| F7 | - | - | - | - | - | - | - | A-C | - | - | - | - | A | - | - | - | - | - | - | - | - | - | - | 2 |
| F3 | - | - | - | - | - | - | - | A-C | - | - | - | - | - | - | - | - | - | - | - | - | T | - | - | 2 |
| F6 | - | A | - | - | - | - | - | A-C | - | - | - | - | - | - | - | - | - | - | - | - | - | - | - | 2 |
| F9 | - | - | - | - | - | - | - | A-C | - | - | - | T | - | - | - | - | - | - | - | - | - | - | - | 2 |
| F10 | - | - | - | - | - | - | - | A-C | T | - | - | - | - | - | - | - | - | - | - | - | - | - | - | 2 |
| F1 | T | - | - | - | - | - | - | A-C | - | - | - | - | - | - | - | - | A | - | - | - | - | - | - | 3 |
| F2 | - | - | - | T | G | - | - | A-C | - | - | - | - | - | - | - | - | - | - | - | - | - | - | - | 3 |
| F11 | - | - | - | - | - | - | - | A-C | - | T | - | - | A | - | - | - | - | - | - | - | - | - | - | 3 |
| F4 | - | - | - | T | G | - | - | A-C | - | - | - | - | - | - | - | - | - | - | - | - | - | A | - | 4 |
| G4 | - | - | - | - | - | - | - | A-C | - | - | A | - | - | A | - | - | - | T | - | G | - | - | - | 5 |
| G7 | - | - | - | - | - | A | - | A-C | - | - | A | - | - | A | - | - | - | - | - | G | - | - | - | 5 |
| G2 | - | - | - | - | - | A | - | A-C | - | - | A | - | - | A | T | - | - | - | - | G | - | - | - | 6 |
| G9 | - | - | - | - | - | - | - | A-C | - | - | A | - | - | A | - | - | - | T | - | G | - | - | T | 6 |
| G0 | - | - | - | - | - | - | T | A-C | - | - | A | - | - | A | - | - | - | T | - | G | - | - | - | 6 |
| G11 | - | - | T | - | - | A | - | A-C | - | - | A | - | - | A | - | - | - | - | - | G | - | - | - | 6 |
| G10 | - | - | - | - | - | - | - | A-C | - | - | A | - | - | A | - | A | - | T | - | G | - | - | T | 7 |
| G8 | - | - | T | - | - | A | - | A-C | - | - | A | - | - | A | - | - | - | - | A | G | - | - | - | 7 |
| G1 | - | - | - | - | - | - | T | A-C | - | - | A | - | - | A | T | - | - | T | - | G | - | - | - | 7 |
| G5 | - | - | - | - | - | A | T | A-C | - | - | A | - | - | A | - | - | - | T | - | G | - | - | - | 7 |
