# Supplementary material for: Multiple origins of melanism in two species of North American tree squirrel (Sciurus)
Source: BMC Evol Biol. 2019 Jul 11;19:140. doi: 10.1186/s12862-019-1471-7 (PMC6625063; doi:10.1186/s12862-019-1471-7)
Supplement: Supplementary file 2 — “Marginal posterior density distributions of parameters from iMa2 analyses.” Graphs showing marginal posterior density distributions of parameters from iMa2 analyses. (PPTX 51 kb) [file 12862_2019_1471_MOESM2_ESM.pptx]

## Slide 1
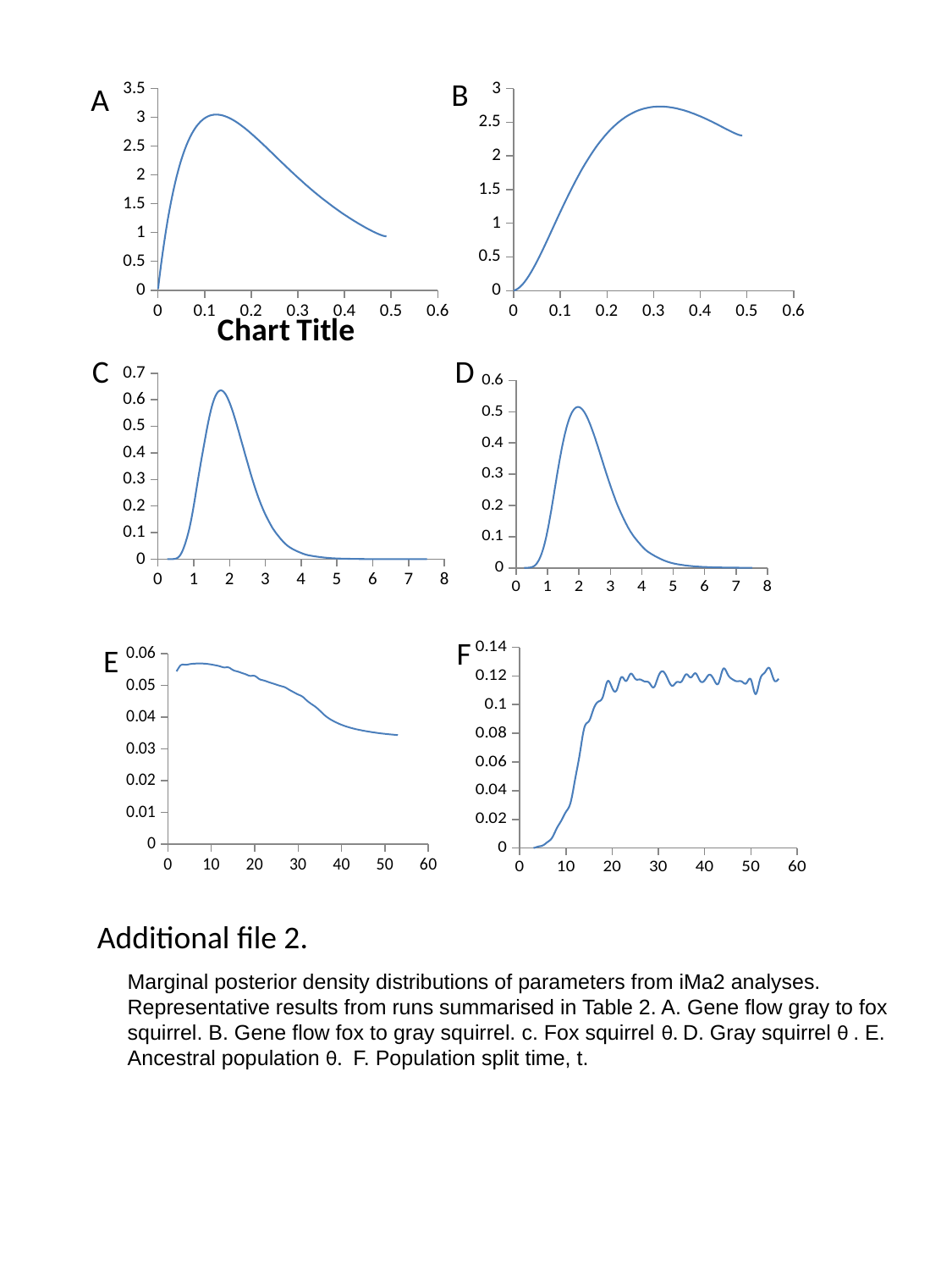

B
A
### Chart
| Category | m0>1 |
|---|---|
### Chart
| Category | m1>0 |
|---|---|
### Chart:
| Category | |
|---|---|C
D
### Chart
| Category | |
|---|---|F
E
### Chart
| Category | |
|---|---|
### Chart
| Category | |
|---|---|Additional file 2.
Marginal posterior density distributions of parameters from iMa2 analyses.
Representative results from runs summarised in Table 2. A. Gene flow gray to fox squirrel. B. Gene flow fox to gray squirrel. c. Fox squirrel θ. D. Gray squirrel θ . E. Ancestral population θ. F. Population split time, t.
