# Supplementary material for: Multiple origins of melanism in two species of North American tree squirrel (Sciurus)
Source: BMC Evol Biol. 2019 Jul 11;19:140. doi: 10.1186/s12862-019-1471-7 (PMC6625063; doi:10.1186/s12862-019-1471-7)
Supplement: Supplementary file 3 — “ASIP haplotypes (coding sequence) from the fox squirrel (A1 – A3, A5, A6) and gray squirrel (A4) with the most common haplotype as reference.” A table showing ASIP haplotypes. (DOCX 12 kb) [file 12862_2019_1471_MOESM3_ESM.docx]

Additional file 3. *ASIP* haplotypes (coding sequence) from the fox squirrel (A1 – A3, A5, A6) and gray squirrel (A4) with the most common haplotype as reference. Bold numbers refer to non-synonymous substitutions, and the substitution leading to the Gly121Cys mutation associated with melanism in haplotype A3 is underlined.

| Base pair/  Allele | **52** | **253** | 360 | **361** |
| --- | --- | --- | --- | --- |
| A1 | T | C | C | G |
| A2 | - | G | - | - |
| A3 | - | G | - | T |
| A5 | - | G | T | - |
| A6 | - | - | T | - |
| A4 | C | - | - | - |
