# Supplementary material for: Multiple origins of melanism in two species of North American tree squirrel (Sciurus)
Source: BMC Evol Biol. 2019 Jul 11;19:140. doi: 10.1186/s12862-019-1471-7 (PMC6625063; doi:10.1186/s12862-019-1471-7)
Supplement: Supplementary file 4 — “Alignment of ASIP protein sequences from diverse rodents and other mammalian taxa.” A table showing an alignment of ASIP protein sequences from diverse rodents and other mammalian taxa. (DOCX 14 kb) [file 12862_2019_1471_MOESM4_ESM.docx]

Additional file 4. Alignment of ASIP protein sequences from diverse rodents and other mammalian taxa. The ten conserved cysteine residues at the carboxy end are highlighted in yellow and the extra cysteine residue in the fox squirrel allele associated with melanism (A3) is highlighted in red.

Sciurus_niger_A1 MDVTRLLLATLLVFLCIFAAYSHLAPEEKPRDDGSLRSNSSMNLLDFPSVSIVALNKKSKKI-SRKEAEKRTRSSK-KKASMKK-VA---RPRTPRPAPCVATRDSCKSPAPACCDPCVSCLCRFFGSVCSCREINRNC

Sciurus_niger_A2 MDVTRLLLATLLVFLCIFAAYSHLAPEEKPRDDGSLRSNSSMNLLDFPSVSIVALNKKSKKI-SRKEAEKRTRSSK-KKASMKK-VA---GPRTPRPAPCVATRDSCKSPAPACCDPCVSCLCRFFGSVCSCREINRNC

Sciurus_niger_A3 MDVTRLLLATLLVFLCIFAAYSHLAPEEKPRDDGSLRSNSSMNLLDFPSVSIVALNKKSKKI-SRKEAEKRTRSSK-KKASMKK-VA---GPRTPRPAPCVATRDSCKSPAPACCDPCVSCLCRFFCSVCSCREINRNC

S.carolinensis_A4 MDVTRLLLATLLVFLCILAAYSHLAPEEKPRDDGSLRSNSSMNLLDFPSVSIVALNKKSKKI-SRKEAEKRTRSSK-KKASMKK-VA---RPRTPRPAPCVATRDSCKSPAPACCDPCVSCLCRFFGSVCSCREINRNC

Ictidomys MDITRLLLATLLVFLCILAAYSHLAPEEEPRDDGSLRSNSSMKVLDIPSVSIVALNKKSQKI-SRTEAEKRTRSSK-KKAPMKK-VA---RPRPPPPTPCVATRDSCKSPSPPCCDPCASCVCRFFGSVCSCRVLNRNC

Castor MDSTRLLLATLLAFLCFLTAYSHLAPEENPRDDKSLRNNSPVNLLDLPSVSIVALNKKSRKI-SRKEAENRKRSSK-KKDSMKK-VA---RPRALLPAPCVATRDSCKPPAPACCDPCASCHCRFFGSTCSCRALNPNC

Mus MDVTRLLLATLVGFLCFFTVHSHLALEETLGDDRSLRSNSSMNSLDFSSVSIVALNKKSKKI-SRKEAEKRKRSSK-KKASMKK-VA-----RPPPPSPCVATRDSCKPPAPACCDPCASCQCRFFGSACTCRVLNPNC

Rattus MDVTRLLLATLVGFLCFLTVHSHLVFEETLGDDRSLKSNSSINSLDFSSVSIVALNKKSKKI-SRKEAEKRKRSSK-KKASIKK-VA-----RPPPPSPCVATRDSCKPPAPACCNPCASCQCRFFGSACTCRVLNPNC

Peromyscus MDVTRLLLATLVGFLCFLAVYSHLVPEETLRDDKSLRTNSSTDCKDFSSVSIVALKKKSKKI-SIQEAEKQKRSSK-KKASIKK-VA-----RPPPPTPCVATRDSCKPPAPACCDPCASCQCRFFRSVCSCRVLNPNC

Microtus MDVTRLLLATLVGFLCFLTVYSHLGPEETLSDDRSLRSNSSVNSLDFSSVSIVALNKKSNRI-SRKEAEKRKRSSK-MNASLKK-EA-----RPPPPTPCVATRDSCKPPAPACCDPCASCQCRFFRSACTCRVLNPYC

Cricetulus MDVTRLLLATLVGFLCFLAIYSHMVPEETLRDDRSLRSNSSMNSLDFSSVSIVALNKKSKKI-NRKEAEKQKRSSK-KKASTKK-VA-----RPPPPTPCVATRDSCKPPAPACCDPCASCQCRFFRSACTCRALNPNC

Jaculus MDVSRLLLATLVVFLCVLTASSHLVH-ENPRDDTSLRSNSSVSLKDFSSVSIVALNKKSKKI-SRKEAEN-KRSSK-KTATTKK-AP---RPRPPPPTPCVATRDSCKPPAPACCDPCASCHCRFFRSACSCRVFNPNC

Cavia MDATRLLLATLLVL-YFLTACSHLPLEEKPKDDRYLRSNSSKNFVDFPSVSIVALNKKSKTF-SKKEAEKRKRSSK-KKASMKE-VK---PPRPPPPAPCVATRDSCKPPAPACCNPCASCQCRFFGSVCSCRVLNLHC

Heterocephalus MDVTRLLLSTLLVFLCFLTACSHLALEEKPKDDRCLRSNSSKNLLDFPSVSIVALNKKSKTI-SRKEAEKRKRSAK-KKASMKKKVA---RPRPPLPEPCVATRDSCKPPAPACCKPCASCQCRFFRSFCSCRVLNPNC

Homo MDVTRLLLATLLVFLCFFTANSHLPPEEKLRDDRSLRSNSSVNLLDVPSVSIVALNKKSKQI-GRKAAEK-KRSSK-KEASMKK-VV---RPRTPLSAPCVATRNSCKPPAPACCDPCASCQCRFFRSACSCRVLSLNC

Microcebus MDVTRLLLATLLVFMCFFTTYSHLPPEEKPRDDRVLRSNSSMNLLDFSSVSIVALNKKFKRI-SRKEAEK-KRSSK-KEASVKK-VA---QPRPPPPAPCVATRFSCKPPAPACCDPCAYCHCRFFRSACSCRVLNPYC

Oryctolagus MNVTRLLQATLLVFLCFLTAYSHLAPEETPTDDQSLRSNSSTNLLEFSSVSIVALNKKSKDI-SIKEAEKKKRSSK-KKASKKK-VA---RPRPLLPAPCVATRDSCKPPAPVCCDPCASCQCRFFRSVCTCRVLNPNC

Tupaia MDVTRLFLAILLVFLYFLTAFSHLAPEEKPREDRRLRSNSSMNLLDFPSVSIVALNKKSKAI-SRKEAEK-KKSSK-KKASMEK-TM---RSRPPPPVPCVATRDSCKPPAPACCDPCAFCQCRFFRSSCTCRVFNPGC

Myotis MDVTRLLLATLLVCLCFLTAYSHLAPEEMPTNDRSLRSNSSMNLLDSPSVSIMGLNKKSKKI-SRKEAEK-KTASKQRKASIKK-VT---RVRPPPPDPCVATRDSCKQPAPPCCDPCASCMCRFFRSTCSCRVLDPNC

Bos MDVSRLLLATLLVCLCFLTAYSHLAPEEKPRDERNLKNNSSMNLLDFPSVSIVALNKKSKKI-SRNEAEKKKRPSK-RKAPMKN-VA---RTRPPPPTPCVATRDSCKPPAPACCDPCAFCQCRFFRSACSCRVLNPTC

Ovis MDVSRLFLATLLVCLCFLSAYSHLAPEEKPRDERNLKNNSSMNLLDFPSVSIVALNKKSKKI-SRNEAEKKKRASK-RKAPMKN-VA---RTRPPPPTPCVATRDSCKPPAPACCDPCAFCQCRFFRSACSCRVLNPTC

Sus MDVTRLLLATLLVCLCFFTASSHLAPEEKSKDERSLRSNSSMNLLDFPSVSIVALNKKSKKI-SRKEAE--KRSSK-KKASMKK-VA---QPRPPRPAPCVANRDSCKPPALACCDPCAFCQCRFFRSACSCRVLNPTC

Canis MNIFRLLLATLLVSLCFLTAYSHLA-EEKPKDDRSLRSNSSVNLLDFPSVSIVALNKKSKKI-SRKEAEK-KRSSK-KKASMKN-VS---HPRPPPPTPCVATRNSCKSPAPACCDPCASCQCRFFRSACTCRVLSPRC

Felis MNILRLLLATLLVCLCLLTAYSHLAPEEKPRDDRNLRSNSSMNMLDLSSVSIVALNKKSKKI-SRKEAEK-KRSSK-KKASMKN-VAQPRRPRPPPPAPCVATRDSCKPPAPACCDPCASCQCRFFRSSCSCRVLNPTC

Ursus MNIFHLLLATLLVSLCFLTAYSHLAPEEKPGDDRSLRSNSSANLLDFPSVSIVALNKKSKKI-SRKEAEK-KRSSK-KKPSRKN-VA---RPRPPPPTPCVATRDSCKPPAPVCCDPCASCQCRFFRSACACRVLRPDC

Equus MDVIHLFLATLLVSLCFLTAYSHLSPEEKPKDDRSLRNNSSMNLLDSPSVSIMALNKKSKKI-SRKEAEKKKRSSK-KKASMTK-VA---RPRLLQPAPCVATRDSCKPPAPACCDPCASCQCRFFRSACSCRVLTRTC

Sorex MTVPRLLLLTLLVFLCGLAAYCHLAPED----DKSLRSNSSLNLLDFPAVSIVALNKKSKNI-SRKEAE--KKSSK-KRASMKK-AA-----RPRLPSPCVATRDSCRPPAPPCCDPCASCQCRFFRSACSCRVTNPNC

Loxodonta MDVTRLFLASLLVCLYFLIAHSHLELKEKPRDDRSLKSNCSMKLLDFPSVSVVALNKKSKRILSRKEAEMMKGPSK-KKASVKK-AA---RPRPPPPAGCVATRDSCKPPAPACCDPCASCQCRFFRSACSCRVLSPNC

Dasypus MDVTRLLLASLLLSLCFLAACSHLAPEDESRDDRSLRSNSSMNLLDFPSISIVAVNKKSERI-SRKEAEK-KKSSK-KKAPVKK-AA---PPPSPPPGPCVATRRSCRPPAPACCDPCASCQCRLFGSACSCRVLSPHC

Monodelphis MTTKHLLLPILLAGLCFLAVYCHLAEEEKWNKDGGLGRNSTLSLPDFPSVSIVALNKKSKKL-IQKEIET-KKSPE-KKALVKK------SHRPPPPANCVATWGNCQPLASPCCNPCAICHCRFFRSVCSCRLFRPRC

Phascolarctos MTSKHLLLLFLLACLWFLAVYCHLAEEEKWSKDKGLGRNSTMNLPDSPSVSIVALNKKSKKI-IRKEIET-KKSSE-KTALVKK------NPRPPPPANCVATWSNCQPLARPCCHPCAMCHCRFFRSVCSCRLFRPRC
